# Embedding Ordered Mesoporous Carbons into Thermosensitive Hydrogels: A Cutting-Edge Strategy to Vehiculate a Cargo and Control Its Release Profile

**DOI:** 10.3390/nano10112165

**Published:** 2020-10-29

**Authors:** Monica Boffito, Rossella Laurano, Dimitra Giasafaki, Theodore Steriotis, Athanasios Papadopoulos, Chiara Tonda-Turo, Claudio Cassino, Georgia Charalambopoulou, Gianluca Ciardelli

**Affiliations:** 1 Department of Mechanical and Aerospace Engineering, Politecnico di Torino, 10129 Turin, Italy; rossella.laurano@polito.it (R.L.); chiara.tondaturo@polito.it (C.T.-T.); gianluca.ciardelli@polito.it (G.C.); 2 PolitoBIOMed Lab, Politecnico di Torino, 10129 Turin, Italy; 3 National Centre for Scientific Research “Demokritos”, 15341 Athens, Greece; d.giasafaki@inn.demokritos.gr (D.G.); t.steriotis@inn.demokritos.gr (T.S.); thapap@rrp.demokritos.gr (A.P.); 4 Department of Science and Technological Innovation, Università del Piemonte Orientale, 15121 Alessandria, Italy; claudio.cassino@uniupo.it

**Keywords:** ordered mesoporous carbons, thermosensitive hydrogels, poly(ether urethane)s, ibuprofen, drug release

## Abstract

The high drug loading capacity, cytocompatibility and easy functionalization of ordered mesoporous carbons (OMCs) make them attractive nanocarriers to treat several pathologies. OMCs’ efficiency could be further increased by embedding them into a hydrogel phase for an *in loco* prolonged drug release. In this work, OMCs were embedded into injectable thermosensitive hydrogels. In detail, rod-like (diameter ca. 250 nm, length ca. 700 nm) and spherical (diameter approximately 120 nm) OMCs were synthesized by nanocasting selected templates and loaded with ibuprofen through a melt infiltration method to achieve complete filling of their pores (100% loading yield). In parallel, an amphiphilic Poloxamer^®^ 407-based poly(ether urethane) was synthesized ($\bar{M_{n}}$ 72 kDa) and solubilized at 15 and 20% *w/v* concentration in saline solution to design thermosensitive hydrogels. OMC incorporation into the hydrogels (10 mg/mL concentration) did not negatively affect their gelation potential. Hybrid systems successfully released ibuprofen at a slower rate compared to control gels (gels embedding ibuprofen as such), but with no significant differences between rod-like and spherical OMC-loaded gels. OMCs can thus work as effective drug reservoirs that progressively release their payload over time and also upon encapsulation in a hydrogel phase, thus opening the way to their application to treat many different pathological states (e.g., as topical medications).

## 1. Introduction

The last few decades have seen a revolutionary era in the field of drug delivery, leading to the development of several innovative cutting-edge approaches for diagnosis and treatment of many pathological states. Among the wide plethora of available approaches, the use of carriers as drug delivery vehicles has received a huge interest, overcoming many drawbacks of the traditional systemic administration route. In summary, drug transportation by specific carriers improves pharmacokinetic profiles, reduces the risk of side effects, and allows a localized and sustained payload release in the target area, with consequent advantages in terms of the required administered dosage to achieve the desired therapeutic effect. In addition, such carriers can be properly engineered to pass through many biological barriers and actively target a specific pathological area of the body. Micro- and nano-particles are among the most widely investigated drug carriers in nanomedicine. They are available in different sizes, forms and materials, such as synthetic polymers, proteins, lipids and inorganic materials. Among them, mesoporous particles hold huge potential in nanomedicine, due to their high versatility, allowing a fine-tuning of their physico-chemical and structural properties, the high loading capacity for many different payloads (e.g., hydrophilic and hydrophobic drugs, growth factors, microRNAs) and their potential for controlled release of their cargo. In particular, the use of mesoporous silica nanoparticles (MSNs) has experienced a great upsurge over the last decade, as recently thoroughly reviewed by Narayan and co-workers [1] and Manzano and Vallet-Regì [2]. For instance, Saini et al. recently reported on the design of MSNs with tunable pore diameter to enhance the delivery of gemcitabine in pancreatic cancer treatment [3], meanwhile Kong et al. developed curcumin-loaded MSNs as a potential cancer therapy with improved stability, anti-oxidant and antitumor activity as compared to the freely administered drug [4]. Moreover, a huge effort is being devoted to the surface functionalization of MSNs with the aim to improve their dispersibility in aqueous media [5], to favor their cellular internalization [6], to provide them with additional features, such as anti-oxidant properties [7] or mucoadhesiveness [8], and to make them able to respond to external stimuli, thus triggering the release of their payload [9,10,11,12,13,14]. A further possibility to functionalize MSNs and afford additional bioactivity consists in doping their framework with other elements, which will be then released in the form of therapeutic ions, such as copper, strontium and calcium species, in a target area of the body [15,16,17,18,19]. 

Among mesoporous materials, another class of particles, namely mesoporous carbon nanoparticles (MCNs), has also revealed interesting properties for drug delivery. Nevertheless, MCNs have been poorly investigated so far, despite their enormous potential and advantages over their silica-based counterparts. Indeed, MCNs combine the characteristic advantages of mesoporous particles with the benefits of carbonaceous materials: (i) favorable structural properties (e.g., high specific surface area and pore volume) for drug loading; (ii) high versatility to allow control over payload release kinetics; (iii) easy surface functionalization for an active targeting or a triggered payload release; (iv) excellent heat conversion capability for potential application in photothermal therapy; (v) high biocompatibility and chemical stability; and (vi) superior loading capacity of aromatic and poorly water soluble compounds [20]. Representing a next generation of mesoporous materials for drug delivery, MCNs are currently experiencing a similar growth, as did MSNs in the past. Whereas MSNs require chemical agents (e.g., silanes, glutaraldehyde) or physical methods (e.g., laser treatment) for their surface functionalization [21,22], abundant functional groups (manly carboxyl groups) can be easily exposed on MCN surface through oxidation treatment [20]. Similar to their silica counterparts, MCNs have also been precisely processed to provide them with specific features. For instance, MCNs have been recently surface functionalized to achieve a pH-triggered payload release [23], decorated with silver nanoparticles to design an anti-bacterial platform for wound healing applications [24], and surface-modified to overcome mucous and epithelial biological barriers, thus improving drug bioavailability [25]. Among the wide variety of mesoporous materials available for drug loading (e.g., MCNs, MSNs, mesoporous hydroxyapatite and mesoporous metallic oxide), the low density, high porosity and strong adsorption ability of MCNs make them able to host huge amounts of drugs (in particular hydrophobic drugs that usually suffer for poor bioavailability), which is a strict requisite for all those applications requiring high drug dosages [20]. In addition, the high MCN drug loading capacity and their capability to generate a photothermal effect are currently under investigation to develop combined chemo-photothermal therapies for a non-invasive treatment of cancer [26].

However, irrespective of their nature, a common issue that characterizes all particulate drug delivery systems is the need to selectively accumulate and retain them in the target tissue for the required time to allow a complete payload release at an effective concentration to exert its therapeutic function. In this regard, the encapsulation of drug-loaded particles into a hydrogel vehicle phase could represent an effective strategy to locally inject a predefined volume of a therapeutic formulation, which will be then in situ retained for a predefined time interval, while progressively releasing the payload. For instance, polymeric particles have been loaded into hydrogels to achieve a sustained and prolonged release of neuroprotective and neuroregenerative drugs [27], growth factors (i.e., stromal cell-derived factor-1, vascular endothelial growth factor) [28], insulin [29,30] and platinum compounds for cancer treatment [31]. MSN-hydrogel hybrid formulations have been also widely explored for the delivery of drugs/biomacromolecules for cartilage regeneration [32], curcumin for dermal application [33], ibuprofen as an anti-inflammatory drug [34,35] and therapeutic ions [35,36]. In addition, we have recently demonstrated that the encapsulation of pH-sensitive MSNs into thermosensitive hydrogels did not alter their responsiveness to external environmental changes (i.e., pH changes in the surrounding environment) [37]. On the contrary, to the best of our knowledge there are no reports in the literature on MCN encapsulation into a hydrogel vehicle phase. Hence, the first aim of the present work was to investigate the loading of two ordered mesoporous carbons (OMCs), which have different geometrical and pore properties into polymeric thermosensitive sol-gel systems based on a customized poly(ether urethane) containing Poloxamer^®^ 407 as a building block [24,35,36,37]. Complete characterization of OMCs was performed through Scanning Electron Microscopy, Small Angle X-ray Scattering, N_2_ adsorption/desorption measurements at 77K, Differential Scanning Calorimetry and Thermogravimetric Analysis, while the successful synthesis of the poly(ether urethane) was verified through Infrared spectroscopy, Nuclear Magnetic Resonance spectroscopy and Size Exclusion Chromatography. Hydrogels were prepared at 15 and 20% *w/v* concentrations and loaded with OMCs at 10 mg/mL concentration. The effects of OMC loading on the gelling properties of the hybrid formulations were studied through qualitative tube inverting tests and rheological analyses. As a second step, the anti-inflammatory drug ibuprofen (IBU) was loaded into the OMCs through a melt infiltration method, leading to IBU-OMCs composites, which were encapsulated into the hydrogels and the resulting formulations were rheologically characterized. Lastly, the release profile of the drug in physiological-like conditions was assessed and compared to that measured from gel systems loaded with pristine ibuprofen molecules at the same concentration.

## 2. Materials and Methods

### 2.1. Synthesis of Ibuprofen-Loaded Ordered Mesoporous Carbons

#### 2.1.1. Materials

The triblock copolymers EO_20_PO_70_EO_20_ (ethylene oxide-propylene oxide-ethylene oxide, Pluronic P123) and EO_106_PO_70_EO_106_ (Pluronic F127), cationic cetyltrimethylammonium bromide (CTAB) and tetraethyl orthosilicate (TEOS 98%) were purchased from Sigma-Aldrich (St. Louis, MO, USA) and used to synthesize mesoporous silicas, which were then employed as templates for OMC synthesis. Sucrose (≥99.5%, Sigma-Aldrich, St. Louis, MO, USA) was used as a carbon precursor during OMC synthesis. Ibuprofen (>98%) was purchased from Sigma-Aldrich (St. Louis, MO, USA). All solvents were also purchased from Sigma-Aldrich (St. Louis, MO, USA) in the analytical grade and used as received.

#### 2.1.2. Rod-Like CMK-3 Type Ordered Mesoporous Carbons

The CMK-3–type carbon material (denoted hereafter as C3) was synthesized, starting from the hexagonally ordered mesoporous silica SBA-15 using a standard hard-templating approach [38]. The SBA-15 silica template was prepared according to a typical method [39], using Pluronic P123 as surfactant agent, and TEOS as a silica source at a composition of 4 g of P123:0.041 mol TEOS:6.67 mol H_2_O:0.24 mol HCl. The hydrothermal reaction took place in an autoclave at 35 °C for 20 h and the aging process was conducted at 90 °C for 24 h. Aiming to obtain a carbon sample with the inverse structure, SBA-15 was impregnated twice with an acidic sucrose solution, subsequently polymerized at 100 °C and 160 °C in air for 6 h and pyrolyzed at 900 °C for at least 2 h in a tube furnace under N_2_ gas flow (100 mL/min). The synthesis was concluded after etching of the silica framework using HF at room temperature (RT) and thorough washing of the remaining carbon with water and ethanol [40].

#### 2.1.3. Spherical CMK-1 Ordered Mesoporous Carbon

In a similar approach, CMK-1 carbon spheres (denoted hereafter as C1) were prepared using sucrose as a carbon precursor and MCM-48 spheres as a starting template. MCM-48 is a 3D cubic periodic silica with an interpenetrating network of pores, in contrast to the uni-dimensional hexagonal pores of SBA-15. Τhe MCM-48 silica spheres were prepared based on a modified Stöber method [41], using a mixture of two surfactants (CTAB as porogen and Pluronic F127 as shape modulator), ethanol, ammonia, H_2_O and TEOS at room temperature [42]. The impregnation, polymerization and pyrolysis conditions were identical to the C3 case. The carbon with the inverse structure and the spherical morphology was obtained again by dissolution of the silica with hydrofluoric acid.

#### 2.1.4. Drug-Loaded Ordered Mesoporous C3 and C1 Carbons

A melt infiltration method was used to incorporate ibuprofen (denoted hereafter as IBU) into the pores of the carbon particles (both C3 and C1). More specifically, C3 or C1 carbon and IBU powders were ground together to obtain a physical mixture with a 1:1 weight ratio, aiming to completely fill the total pore volume of the particles. The mixture was immersed in a water bath at 86–87 °C, i.e., above the melting point of IBU (75–78 °C), under rotation for 30 min, using a rotary evaporator (RII, BÜCHI AG, Flawil, Switzerland), so that the drug would melt and infiltrate the particles’ pores. Subsequently, the mixture was transferred to an ice bath and was kept there for 10 min to induce the solidification of IBU.

### 2.2. Characterization of C3 and C1 Based Materials

The surface morphology of the synthesized C3 and C1 carbon materials was studied through Scanning Electron Microscopy (SEM) using a JSM 7401F Field Emission Microscope (JEOL Ltd, Tokyo, Japan) equipped with a Gentle Beam mode.

The ordered pore structure of the samples was investigated using Small Angle X-ray Scattering (SAXS) in transmission mode on a SmartLab X-ray diffraction system (Rigaku Corporation, Tokyo, Japan) equipped with SAXS optics (λ = 1.54 Å). The scans were obtained from 0.06 to 8 degrees, with a speed of 0.1 deg/min and a step of 0.02 degrees.

The incorporation of IBU into the carbons’ pores was checked by Thermo-Gravimetric Analysis (TGA) and Differential Scanning Calorimetry (DSC). The measurements were performed on approximately 10 mg of each drug-loaded carbon sample, in the range of 25–600 °C, with a heating rate of 10 °C/min in an Al_2_O_3_ crucible under Argon flow (30 mL/min), using a SETSYS Evolution 18 Analyser (Setaram Instrumentation, Caluire-et-Cuire, France). Purging prior to thermal analysis as well as buoyancy corrections through blank measurements were also carried out.

The pore properties of the plain and the IBU-loaded carbons were evaluated by N_2_ adsorption/desorption measurements at 77 K on a volumetric gas adsorption analyzer (Autosorb-1-MP, Quantachrome Inc., FL, Boynton Beach, USA), using ultra-pure (99.999%) N_2_. Before analysis, the samples (approximately 30–40 mg) were appropriately outgassed for at least 20 h under high vacuum (10^−6^ mbar). The Brunauer-Emmett-Teller (BET) area values were calculated on the basis of the BET consistency criteria (ISO 9277:2010). The micropore volumes were assumed to be the QSDFT (Quenched Solid Density Functional Theory)-derived cumulative volumes for pores smaller than 2 nm. The total (micro- and meso-pore) volumes (TPV) were estimated at *p*/*p_0_* = 0.90, whereas the pore size distributions were deduced by using the N_2_-carbon QSDFT kernel for slit-cylindrical pores (adsorption model).

### 2.3. Synthesis of Poly(Ether Urethane)

#### 2.3.1. Materials

The poly(ether urethane) (PEU) used in this work was synthesized using the commercially available triblock copolymer Poloxamer^®^ 407 (poly(ethylene oxide)-poly(propylene oxide)-poly(ethylene oxide) PEO-PPO-PEO, 12,600 Da, 70% PEO content) as macrodiol, the aliphatic diisocyanate 1,6-hexamethylene diisocyanate (HDI) and the aliphatic cyclic diol 1,4 cyclohexanedimethanol (CDM) as chain extender. All these three building blocks were purchased from Sigma-Aldrich (Milan, Italy) and used after drying/purification procedures. In detail, P407 was anhydrified under reduced pressure at 100 °C overnight and then equilibrated under vacuum at 40 °C until use, HDI was distilled under vacuum to remove moisture and stabilizers and CDM was stored at RT under reduced pressure in a desiccator until usage. Dibutyltin dilaurate (DBTDL) was also purchased from Sigma-Aldrich (Milan, Italy) and used as received to catalyze the PEU synthesis reaction. Anhydrified 1,2-dichloroethane (DCE) was prepared by storing DCE (Carlo Erba Reagents, Cornaredo, Milan, Italy) over activated (120 °C, atmospheric pressure, overnight) molecular sieves (3 Å, Sigma-Aldrich, Milan, Italy) overnight under inert conditions (i.e., nitrogen flow). All other solvents required for PEU synthesis were purchased from Carlo Erba Reagents (Cornaredo, Milan, Italy) in the analytical grade and used as received. All glassware used for PEU synthesis was stored in an oven at 120 °C until use.

#### 2.3.2. Poly(Ether Urethane) Synthesis Protocol

The poly(ether urethane) used in this work to design hybrid sol-gel systems embedding OMCs was synthesized according to an already published protocol [43]. Briefly, the synthesis was conducted in two-steps under inert conditions. Initially, a P407 solution in anhydrous DCE was prepared at 20% *w/v* concentration and equilibrated at 80 °C. Then, HDI (2:1 molar ratio with respect to P407) and a catalytic amount of DBTDL (0.1% *w*/*w* with respect to P407) were added to start the prepolymerization step, which lasted 150 min at 80 °C. At the end of the first step, the polymerization system was cooled down to 60 °C and a CDM solution previously prepared in anhydrous DCE (3% *w/v* concentration) was added to initiate the chain extension reaction. After 90 min, the system was equilibrated at RT, MeOH was added to passivate potential unreacted isocyanate groups and the synthesized PEU was finally collected by precipitation in petroleum ether at 4:1 volume ratio with respect to the whole DCE volume used during the synthesis. After overnight drying under the fume hood, purification was conducted by precipitating a PEU-concentrated solution prepared in DCE in a mixture of diethyl ether/MeOH (98:2 *v/v*, 5:1 volume ratio with respect to DCE). The purified PEU was then collected through centrifugation (MIKRO 220R, Hettich, Tuttlingen, Germany, 20 min, 6000 rpm, 0 °C), dried under the fume hood overnight and finally stored under an inert atmosphere until usage.

Hereafter, the synthesized PEU will be referred to with the acronym CHP407, where C, H and P407 refer to CDM, HDI and Poloxamer^®^ 407, respectively.

### 2.4. Chemical Characterization of as-Synthesized Poly(Ether Urethane)

The successful synthesis of CHP407 poly(ether urethane) was first assessed through its chemical characterization by means of Size Exclusion Chromatography (SEC), Proton and Carbon Nuclear Magnetic Resonance (^1^H and ^13^C NMR, respectively) spectroscopy and Attenuated Total Reflectance Fourier Transformed Infrared (ATR-FTIR) spectroscopy.

SEC analysis was conducted using an Agilent Technologies 1200 Series (Agilent Technologies Inc., Santa Clara, CA, USA) equipped with a Refractive Index Detector (RID) and two Waters Styragel columns (HR4 and HR1), both equilibrated at 55 °C. A LiBr (Sigma-Aldrich, Milan, Italy) solution in N,N-dimethylformammide (DMF, HPLC grade, Carlo Erba Reagents, Cornaredo, Milan, Italy) (0.1% *w/v*) was used as eluent for the analysis at a 0.5 mL/min flow rate. The analyzed sample was prepared by filtering the CHP407 solution (2 mg/mL concentration in the eluent phase) with a syringe filter (0.45 μm pore size, polytetrafluoroethylene membrane, Carlo Erba Reagents, Cornaredo, Milan, Italy). As a result of the chromatographic analysis, the RID signal was registered as a function of elution time. Starting from these data and a calibration curve based on poly(methyl methacrylate) standards (Number Average Molecular Weight ranging between 940 and 214,600 Da), the Agilent ChemStation software finally estimated the CHP407 molecular weight distribution profile and its characteristic parameters, namely the Number and Weight Average Molecular Weight values ($\bar{M_{n}}$ and $\bar{M_{w}}$, respectively) and the polydispersity index ($D=\;\bar{M_{w}}/\bar{M_{n}}$).

^1^H and ^13^C NMR spectra of synthesized CHP407 were recorded using a Bruker Avance NEO instrument (Bruker Italia S.r.l., Milan, Italy) equipped with a 11.74 T magnet (500 MHz ^1^H Larmor Frequency) and a Bruker SmartProbe (Bruker Italia S.r.l., Milan, Italy). Analyses were conducted at 300 K on CHP407 samples prepared using d6_DMSO as solvent. ^1^H and ^13^C NMR spectra resulted from 32 and 10,000 scans, respectively. Registered spectra were then analyzed using MestReNova software (https://mestrelab.com/, Mestrelab Research S.L., Santiago de Compostela, Spain) by referring to the residual d6-DMSO proton and carbon signals at 2.5 and 39.5 ppm, respectively, for chemical shift scale.

ATR-FTIR analysis was conducted on both CHP407 and P407 powder using a Perkin Elmer Spectrum 100 instrument (Perkin Elmer, Waltham, MA, USA) equipped with a diamond crystal (UATR KR55, Perkin Elmer, Waltham, MA, USA) ATR set-up. ATR-FTIR spectra of the polymers were registered at RT and resulted from 16 scans recorded between 4000 and 600 cm^−1^ wavenumber range, with a resolution of 4 cm^−1^. ATR-FTIR spectra of the samples were then analyzed and their characteristic peaks were identified using Spectrum software (Perkin Elmer, Waltham, MA, USA).

### 2.5. Design and Characterization of Hybrid Sol-Gel Systems Based on Thermosensitive PEU Hydrogels and OMCs

#### 2.5.1. Hydrogel Preparation Protocol

CHP407-based sol-gel systems were prepared at a final polymer concentration of 15 and 20% *w/v*. These compositions were selected based on previous works where we reported the complete sol-to-gel transition curve of thermosensitive hydrogels based on PEUs with chemical composition similar to CHP407 [44,45]. Indeed, formulations at 15 and 20% *w/v* PEU concentration have been reported to exhibit fast gelation in physiological conditions, injectability and prolonged stability in aqueous environments. Hybrid PEU/OMC formulations were prepared with a similar approach to the one we have already described [35,36,37], i.e., by mixing a PEU aqueous solution with an OMC aqueous dispersion to achieve a final PEU concentration of 15 or 20% *w/v* and an OMC content of 10 mg/mL. Briefly, the required amount of CHP407 powder was weighed and dissolved at 4 °C overnight in a volume of physiological solution (0.9% NaCl) corresponding to 90% of the total volume required to solubilize it at a 15 or 20% *w/v* concentration. Then, the required amount of each OMC was dispersed in a volume of double demineralized water containing sodium dodecyl sulfate (SDS, 1% *w/v*, Sigma-Aldrich, Milan, Italy) corresponding to 10% of the total volume required to achieve a PEU concentration of 15 or 20% *w/v* in the final hybrid formulations. OMC dispersion was then added to CHP407 samples kept in the sol state in a water bath and the resulting formulations were mixed with a vortex to homogeneously disperse the particles. For instance, in a typical experimental set-up, 150 mg of CHP407 were solubilized in 900 µL of physiological solution. Then, 10 mg of OMCs were weighed and dispersed in an SDS aqueous solution (100 µL, 1% *w/v*), which was then mixed with CHP407 solution to obtain a hybrid sol-gel system with CHP407 and OMCs at 15% *w/v* and 10 mg/mL concentration, respectively. Similar samples embedding OMCs previously loaded with IBU (OMC_IBU) were also prepared.

For comparison, pure CHP407 sol-gel systems were prepared at 15 and 20% *w/v* concentration in an aqueous medium consisting of a mixture of physiological solution and SDS solution (1% *w/v* in double demineralized water) at 90:10 volume ratio.

Table 1 summarizes the compositions and acronyms of the formulations designed and characterized in this work.

#### 2.5.2. Hybrid Hydrogel Characterization

The capability of CHP407-based hybrid formulations to undergo a temperature-driven sol-to-gel transition was assessed through tube inverting test and rheological characterization.

A tube inverting test was performed to estimate the gelation temperature (i.e., the Lower Critical Gelation Temperature, LCGT) of the developed sol-gel systems. To this aim, all the investigated formulations (1 mL) were prepared in Bijou sample containers (Thermo Scientific™ Sterilin™, Waltham, MA, USA ) with 17 mm internal diameter to avoid results dependence over sample geometry and volume. Samples were prepared according to the previously described protocol and subjected to a controlled temperature increase from 4 to 40 °C, at 1 °C/step; at each step the temperature was kept stable for 5 min and was followed by vial inversion and visual inspection for 30 s. Conditions of sol and gel were defined based on the presence of a flow along vial walls during sample inversion. The temperature characterized by the complete absence of flow during the 30 s of vial inversion was defined as the LCGT of the formulation. A tube inverting test was also conducted at a constant temperature of 37 °C to estimate the time required by the samples to undergo complete gelation at physiological temperature. To this aim, samples were incubated at 37 °C and the vials were inverted every 1 min to assess the presence or absence of sample flow along their walls. The incubation time at which no sample flow was detected defined the time required for the investigated formulation to undergo complete sol-to-gel transition at 37 °C.

CHP407-based formulations were also characterized through rheological analyses (MCR302, Anton Paar GmbH, Graz, Austria, parallel plate geometry 50 mm, Peltier system for temperature control). First, strain sweep tests were performed at 37 °C and 10 Hz within the strain range from 0.01 to 500% to characterize the gels’ capability to withstand an applied deformation, and define their linear viscoelastic (LVE) region (i.e., the strain range characterized by a constant value of the measured storage modulus) and Yield Stress (YS, i.e., the value of shear stress at the maximum of loss modulus). The progressive gel formation with an increasing temperature was monitored through temperature ramp tests within the temperature range from 0 to 40 °C, at 2 °C/min and 0.1 Hz. In parallel, the evolution of the sample network from the sol to the gel phase, encompassing the biphasic state, was studied through frequency sweep tests, which were carried out at three different temperatures (i.e., 25, 30 and 37 °C) and strain within the previously defined LVE region, within the angular frequency range from 0.1 to 100 rad/s. Samples were loaded on the lower plate of the instrument equilibrated at 0 °C, then the gap was fixed at 0.6 mm and the system was heated at the testing temperature and left to equilibrate for 10 min before the beginning of the analysis (for temperature ramp tests, equilibration was performed at 0 °C).

### 2.6. Ibuprofen Release from Hybrid Sol-Gel Systems

In order to assess the capability of CHP407/OMC hybrid formulations to work as a reservoir of drugs (e.g., ibuprofen) and progressively release it in the surrounding aqueous environment, IBU release tests were conducted in physiological-like conditions, i.e., at 37 °C and using Phosphate Buffered Saline (PBS, pH 7.4) as a releasing medium. CHP407/OMC hybrid samples (1 mL) were prepared according to the previously described protocol by embedding IBU-loaded OMCs (C1_IBU and C3_IBU) at a final carbon concentration of 1 mg/mL (samples CHP407_15%_C1_IBU_1 and CHP407_15%_C3_IBU_1). For comparison purposes, CHP407-based hydrogels (1 mL) with the same polymer content and loaded with IBU as such at the same concentration as that of CHP407/OMC hybrid samples were prepared according to Boffito and Pontremoli et al. [35] (CHP407_15%_IBU).

All investigated formulations were first incubated at 37 °C in an incubator (LabTech, Sorisole, Italy) for 15 min to allow their complete gelation; then 1 mL of PBS (kept at 37 °C in the incubator to equilibrate) was added to each sample and the release tests started. The release medium was then collected and completely refreshed at 3 h, 24 h, 3 d, 5 d, 7 d, 10 d, 14 d and 21 d incubation times. Collected release media were analyzed through spectrophotometric analysis (UV-1800 spectrophotometer, Shimadzu Corporation, Kyoto, Japan) within the spectral range 400–190 nm by analyzing the IBU absorbance peak at 222 nm. Released ibuprofen was finally quantified by referring to a calibration curve based on IBU standard samples prepared by solubilizing the drug in PBS at concentrations within the range 2.5–200 µg/mL.

### 2.7. Statistical Analysis

Ibuprofen release tests were conducted in triplicate and results are reported as mean ± standard deviation. GraphPad Prism software (GraphPad Software, Inc., La Jolla, CA, USA, version 5.03, 2009; http://www.graphpad.com) was employed to subject ibuprofen release data to a Two-way ANOVA analysis followed by Bonferroni’s multiple comparison test.

## 3. Results

### 3.1. Physico-Chemical Characterization of Pristine and IBU-Loaded OMCs

#### 3.1.1. Structural Characterization of Pristine C1 and C3 Carbons

**Figure 1:**
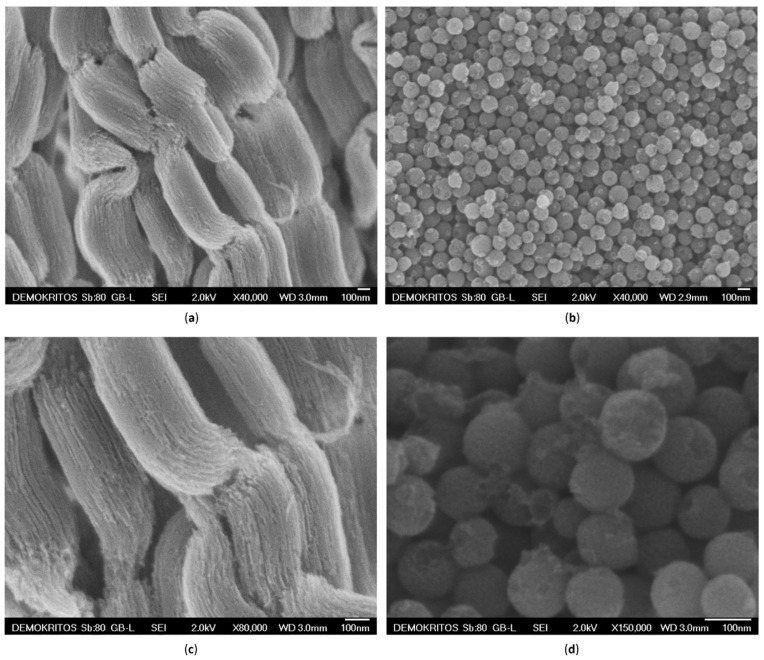
Scanning Electron Microscopy (SEM) images of as synthesized C3 (**a,c**) and C1 (**b,d**).

As shown in the SEM micrographs of Figure 1a,c, C3 carbon exhibited a cylindrical morphology of agglomerated elongated particles of submicron size consisting of highly ordered arrays of carbon nanorods. In detail, the C3 particles showed an average diameter of ca. 250 nm and a length of ca. 700 nm. On the other hand, C1 exhibited a spherical morphology of mono-dispersed nanoparticles, with an average size of approximately 120 nm (Figure 1b,d).

**Figure 2:**
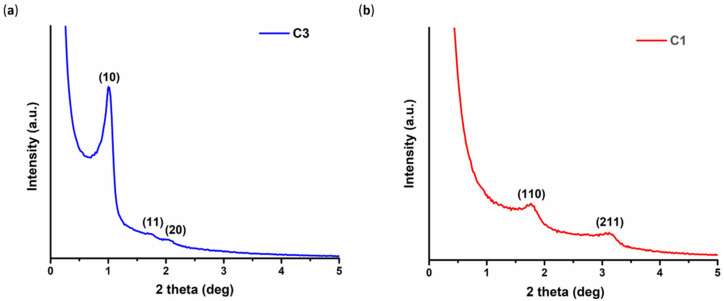
SAXS patterns of the pristine carbons: (**a**) C3 and (**b**) C1.

SAXS diffractograms of the ordered mesoporous carbons C3 and C1 are presented in Figure 2 and they are typical for CMK-3 and CMK-1 structures, respectively, confirming the successful synthesis of the OMCs. In more detail, as shown in Figure 2a, the pattern of C3 depicts three well-resolved diffraction peaks ascribed to the (10), (11), and (20) reflections of the 2D hexagonal ordered arrangement with space group *p6mm^3^*, while the pattern of C1 (Figure 2b) exhibits two well-defined peaks attributed to the (110) and (211) reflections of the 3D tetragonal structure with *I4_1_/a* symmetry [42,46].

#### 3.1.2. Ibuprofen Loading into C1 and C3 Carbons

**Figure 3:**
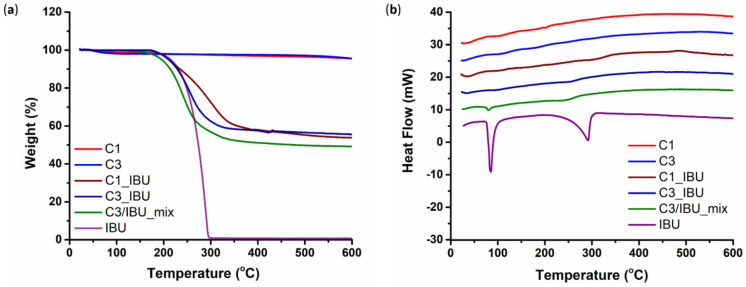
Thermo-Gravimetric Analysis (TGA) (**a**) and Differential Scanning Calorimetry (DSC) (**b**) results of drug-loaded carbons (C1_IBU and C3_IBU), pure ibuprofen (IBU), pristine carbons and a physically mixed C3-IBU sample (C3/IBU_mix) not subjected to prior melt infiltration. Each DSC thermogram was shifted by ca. 5 mW for clarity.

The successful incorporation of IBU within the pores of C3 and C1 carbons was evaluated through TGA and DSC performed on IBU-loaded carbon particles, pure IBU and plain C3 and C1 carbons as references. TGA results (Figure 3a) revealed that the initial 1:1 weight ratio of the carbon/IBU physical mixture was maintained after melt infiltration in both C3 and C1. Indeed, after the decomposition of IBU at around 300–400 °C, the total mass of the drug loaded C3 and C1 decreased by approximately 50% of the initial weight, in agreement with the 1:1 initial loading. DSC analyses (Figure 3b) confirmed that IBU rather resided inside the pores of the carbon materials, and not on their outer surface. As shown in the thermograms of the drug-loaded carbons, no peaks were observed near the melting point of crystalline IBU (70–80 °C), indicating the absence of the crystalline drug. Given that IBU is considered to be in an amorphous and not a crystalline form when confined in pores [47,48], these results provided evidence of the successful infiltration of IBU into the carbons’ pores. Likewise, the decomposition peak (around 280 °C) is missing from the infiltrated samples, while the process is much slower and more gradual compared with the sudden boil-off of the “free” IBU. This may imply that due to Van der Waals interactions between the carbon pore walls and IBU molecules, pore confinement has an additional “drug protection” effect.

Thermal analysis of pristine carbons revealed a minor steady weight loss (Figure 3a,) that could be attributed to the removal of some functional groups on the carbons’ surface. However, this amount may be considered practically negligible when compared to the weight loss resulting from IBU as such. The same measurements (Figure 3a,) were performed also on a C3/IBU physical mixture that had not been subjected to prior melt infiltration (sample acronym: C3/IBU_mix). The obtained DSC pattern (Figure 3b) was similar to the melt infiltrated samples; this was expected, since in a sense, infiltration also occurs in-situ along with melting during the thermal analysis measurements. However, the occurrence of a small peak in the region of the melting point of crystallized IBU suggests that melting is faster than infiltration in a way that the system was able to partially capture the individual melting process of a limited external IBU quantity.

#### 3.1.3. Pore Properties of Plain and IBU-Loaded C1 and C3 Carbons

**Figure 4:**
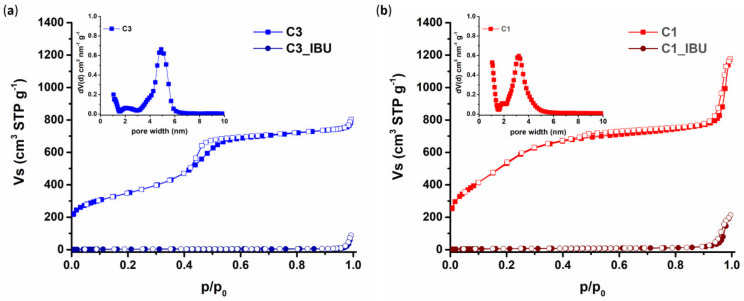
N_2_ adsorption–desorption isotherms (colored and white symbols, respectively) and pore size distribution (PSD) (insets) of pristine and IBU-loaded samples for C3 (**a**) and C1 (**b**) carbons.

The pore properties of the C3 and C1 pristine carbons and their drug-loaded derivatives were assessed by N_2_ adsorption–desorption measurements at 77 K; the respective isotherms along with the corresponding pore size distributions are presented in Figure 4. The isotherms of the C3 and C1 materials were of type IV (based on IUPAC classification [49]), as typical of mesoporous materials. At low relative pressures (*p*/*p_0_* < 0.01), both carbon samples revealed enhanced N_2_ adsorption, indicating the presence of a significant amount of microporosity. Meso- to macro-porosity was also evident at high relative pressures as shown by an increase of the adsorbed amount of nitrogen. In the case of the C3 sample, this increase was not that significant, while in the case of C1, the packing of the carbon spheres led to a large external surface area and a considerable secondary pore volume (the volume of pores with diameters between 20–200 nm is 0.06cm^3^/g for C3 and 0.6 cm^3^/g for C1).

The textural properties of the plain carbon samples are summarized in Table 2. As also presented in the pore size distribution plots (insets of Figure 4), both carbons exhibited a high degree of uniformity of small mesopores, approximately 4.5 nm in size in the case of C3 and approximately 3 nm in the case of C1. On the other hand, both IBU-loaded carbons exhibited isotherms of type II (Figure 4), which are typical of non-porous and/or macroporous materials. In addition, the BET area and the total pore volume of the materials decreased significantly after the incorporation of IBU, as is also shown in Table 2, indicating complete pore filling with the drug (and thus maximum loading).

### 3.2. Chemical Characterization of Synthesized Poly(Ether Urethane)

**Figure 5:**
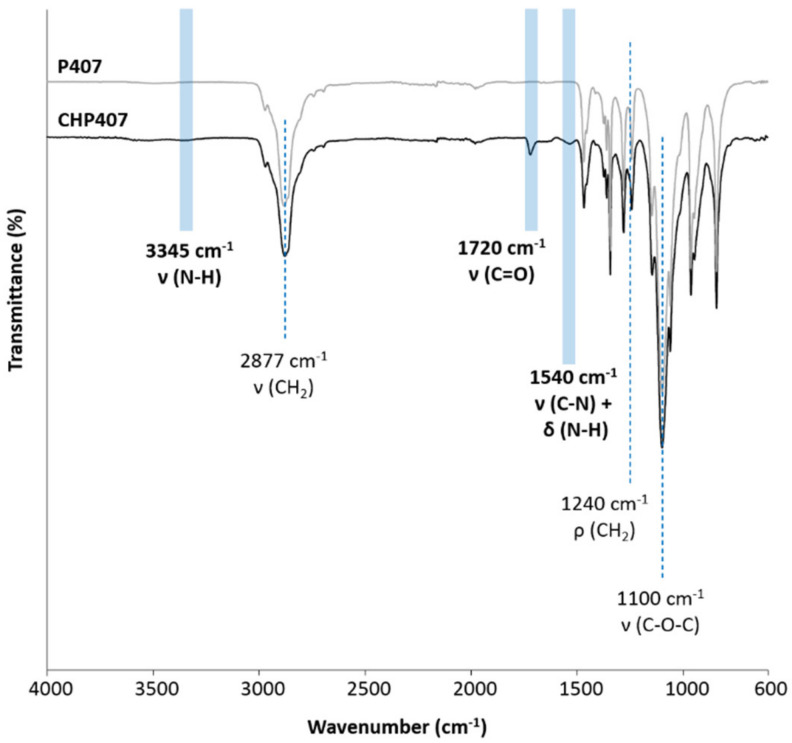
Attenuated Total Reflectance Fourier Transformed Infrared (ATR-FTIR) spectrum of synthesized CHP407 vs. P407. Characteristic peaks proving PEU successful synthesis are identified with light blue bars, while P407 typical signals are highlighted with blue dashed lines.

ATR-FTIR spectroscopy, ^1^H and ^13^C NMR spectroscopy and SEC were conducted on CHP407 powder to demonstrate its successful synthesis. Figure 5 reports the recorded ATR-FTIR spectrum of CHP407. P407 powder was also analyzed for comparison purposes. A visual comparison between CHP407 and P407 ATR-FTIR spectra immediately evidenced the appearance of additional peaks in PEU registered spectrum compared to that of the macrodiol used for its synthesis, namely P407. In more detail, new absorbance peaks appeared in CHP407 spectrum at 3345 cm^−1^, ascribable to the stretching vibration of N-H bonds, at 1540 cm^−1^, due to the simultaneous bending of N-H and the stretching of C-N bonds, and at 1720 cm^−1^ resulting from the stretching of carbonyl groups of newly formed urethane bonds [43,50]. The appearance of these signals in the CHP407 spectrum thus proved the successful formation of urethane bonds. In addition, the presence of typical peaks of P407 (at 2877, 1100, and 1240 cm^−1^) proved its inclusion in the PEU backbone. Finally, the absence of absorbance bands at ca. 2270 cm^−1^ demonstrated the complete conversion of available isocyanate groups.

**Figure 6:**
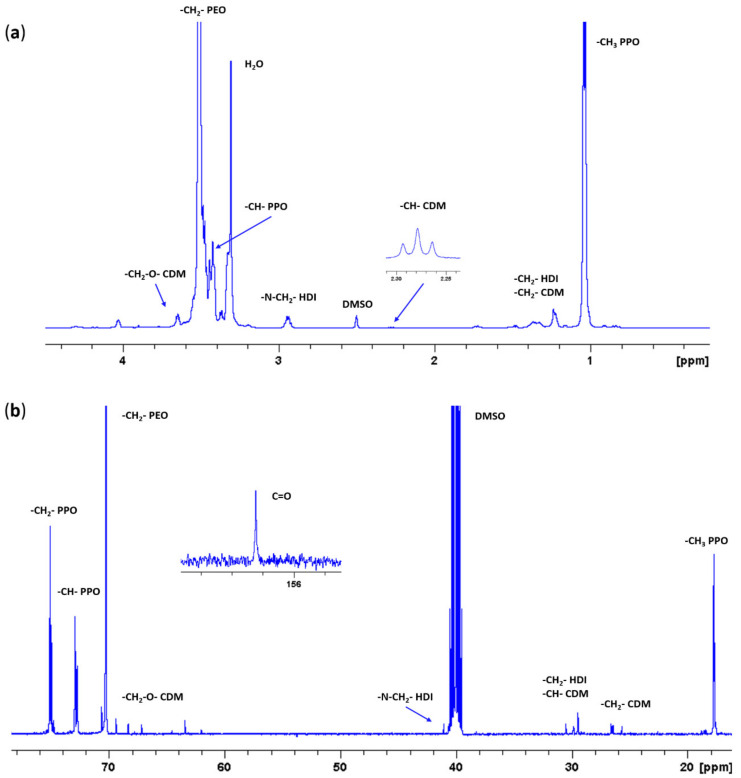
(**a**) Proton (^1^H) and (**b**) Carbon (^13^C) Nuclear Magnetic Resonance (NMR) spectra of CHP407.

Figure 6 reports the recorded ^1^H and ^13^C NMR spectra of as synthesized CHP407, which further corroborated ATR-FTIR spectroscopy results. Indeed, in both the spectra the typical signals of PEU building blocks (i.e., PEO and PPO blocks of P407, HDI and CDM) were evident. Moreover, in the ^1^H NMR spectrum, the resonance of the HDI methylene protons adjacent to the urethane bonds (i.e., -N-CH_2_- of HDI) appeared at ca. 2.93 ppm, meanwhile the signal ascribable to the same group was present at around 41 ppm in the ^13^C NMR spectrum. A further demonstration of the successful CHP407 synthesis was provided by the appearance of the urethane carbonyl group resonance at ca. 156.6 ppm in the ^13^C NMR spectrum.

Finally, SEC analysis of CHP407 revealed a Number Average Molecular Weight ($\bar{M_{n}}$) of 72 kDa and a polydispersity index of 1.7.

### 3.3. Characterization of Hybrid Sol-Gel Systems Based on Thermosensitive PEU Hydrogels and OMCs

#### 3.3.1. Tube Inverting Test

**Figure 7:**
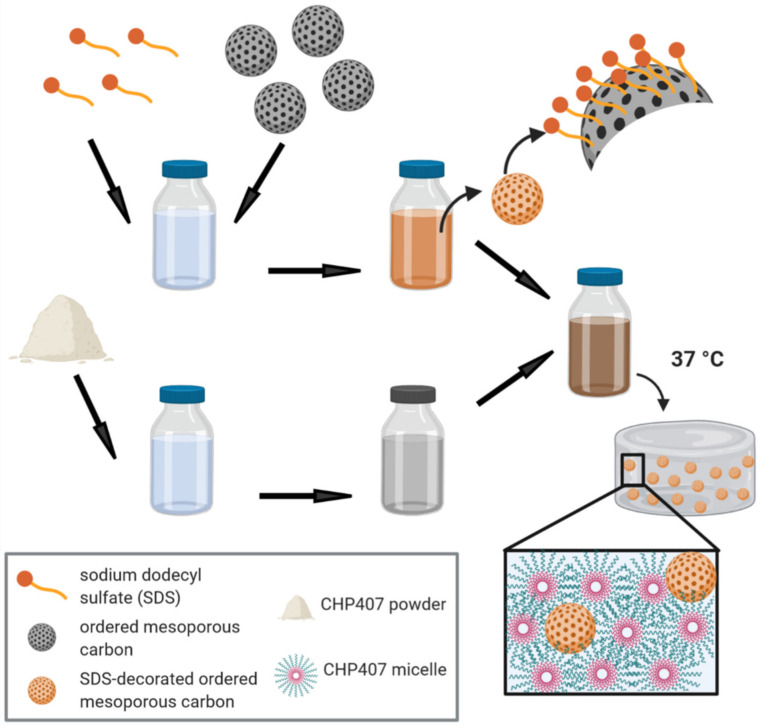
Schematic representation of the process of hybrid hydrogel preparation and the resulting gel network containing carbon nanoparticles. First Ordered Mesoporous Carbons (OMCs) (gray particles in the figure) are dispersed in a Sodium Dodecyl Sulfate (SDS) aqueous solution resulting in an SDS-surface decorated ordered mesoporous carbons (orange particles in the figure). In parallel, the required amount of CHP407 powder is solubilized in physiological solution overnight at a low temperature. The two components are finally mixed together, leading to hybrid hydrogels which result in gel networks homogeneously entrapping SDS-decorated OMCs upon heating at 37 °C. This figure was created with BioRender.com.

The principle of tube inverting test was utilized for a preliminary investigation of the effects of OMC encapsulation on the gelation temperature and time of CHP407-based sol-gel systems. Carbon-free CHP407 samples were also characterized as reference. The gelation temperature of CHP407_15% and CHP407_20% hydrogels slightly decreased upon OMC loading, with no differences induced by the different morphologies of C1 and C3. For instance, CHP407_15% sample decreased its gelation temperature from 27 °C to 25 °C upon addition of C1 or C3 at 10 mg/mL concentration. Similarly, CHP407_20% and OMC-containing CHP407_20% hybrid formulations exhibited gelation temperature at 22 °C and 20 °C, respectively. Despite the qualitative nature of the method and its characteristic error of ± 0.5 °C, the tube inverting test suggested that the inclusion of OMCs into thermosensitive hydrogels slightly decreased their gelation temperature, in disagreement with our previously reported results on CHP407 hybrid formulations loaded with inorganic mesoporous bioactive glasses with a similar dimension [36]. The differences could be correlated to the presence of the surfactant SDS into the hydrogel formulations. Indeed, SDS molecules were expected to interact with the OMC surface through their tail, thus resulting in SDS-coated particles with the potential to better disperse into a polymeric gelling phase and physically interact with it (Figure 7). In addition, such particles exposing SDS polar head to the surrounding environment were most likely involved in the formation of hydrogen bonds with CHP407 micelles, which have been reported to synergistically contribute together with hydrophobic interactions to the chain aggregation phenomenon and the consequent sol-to-gel transition [51]. Altogether, these considerations could explain the observed differences with our previous work [36] and are in accordance with recently published observations by our group on the micellization and gelation process of amphiphilic polymer chains bearing carboxylic acid or primary amino groups along their backbone [37,43].

#### 3.3.2. Rheological Characterization

**Figure 8:**
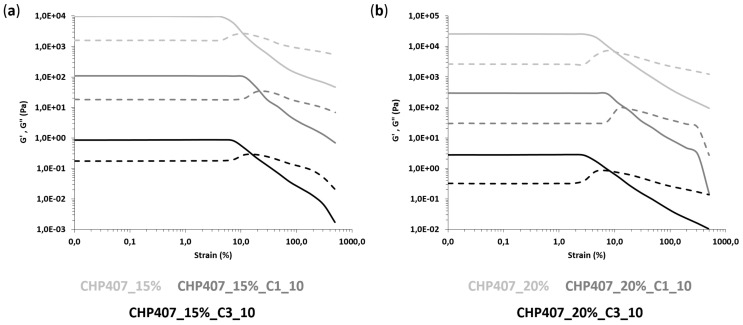
Trends of storage (G’, continuous line) and loss (G’’, dashed line) moduli as a function of applied deformation for CHP407 hydrogels at (**a**) 15% *w/v* and (**b**) 20% *w/v* as such and upon addition of C1 and C3 particles at 10 mg/mL concentration. G′ and G″ values of C1- and C3-loaded hydrogels were divided by a factor of 100 and 10,000, respectively.

In order to obtain a further insight into the gelation process of OMC-loaded sol-gel systems, rheological characterization was performed through oscillatory tests, namely strain and frequency sweep tests, as well as rotational temperature ramp tests. Hydrogels without particles were also characterized as a control condition. First, strain sweep tests were conducted to characterize the gel response to the applied deformation. Figure 8 and Table 3 report the trends of storage (G’) and loss (G’’) moduli as a function of the applied strain and the characteristic parameters (i.e., critical deformation -γ_L_-, yield stress -YS- and the G’-G’’ delta within the LVE -Δ_G’-G’’_-) of all the investigated samples, respectively.

All gels exhibited an LVE region that is a strain range showing constant storage and loss moduli values. The upper limit of the LVE region defined a critical strain value (γ_L_) corresponding to the application of a percentage of deformation, which induced the appearance of cracks into the gel network. For an applied strain higher than γ_L_, storage modulus decreased, meanwhile the loss modulus trend initially increased due to a strain hardening response of the samples and then decreased due to the complete failure of the systems. As a matter of fact, whereas the samples initially behaved as gel systems, being G’ values higher than the G’’ ones, starting from the G’/G’’ crossover, they started to reply to applied deformation as typical of solutions, with G’’ becoming higher than G’.

OMC-loading into CHP407 hydrogels turned out to affect hydrogel response to applied deformation. Indeed, at the same polymer and particle concentration (i.e., 15 or 20% *w/v* CHP407 concentration, and 10 mg/mL particle concentration), the loading of either C1 or C3 resulted in an increase of γ_L_, Δ_G’-G’’_ and YS, with much more relevant changes in the case of C1 hybrid formulations (approximately a 2.5-fold increase in γ_L_ and a 2-fold increase in YS). This result is in agreement with the previously hypothesized role exerted by SDS terminal heads exposed on OMC particle surfaces. The presence of hydrogen bonds among SDS heads and the polymer chains resulted in the formation of stronger networks, with improved mechanical properties and resistance to the applied strain. The observed differences between C1 and C3 particles could be ascribed to their different geometrical features: the higher external surface area exposed by C1 (due to its spherical shape and smaller particle size) accounted for better interactions with CHP407 chains via the SDS molecules coating them and induced the introduction of smaller and well-integrated defects into the gel network.

**Figure 9:**
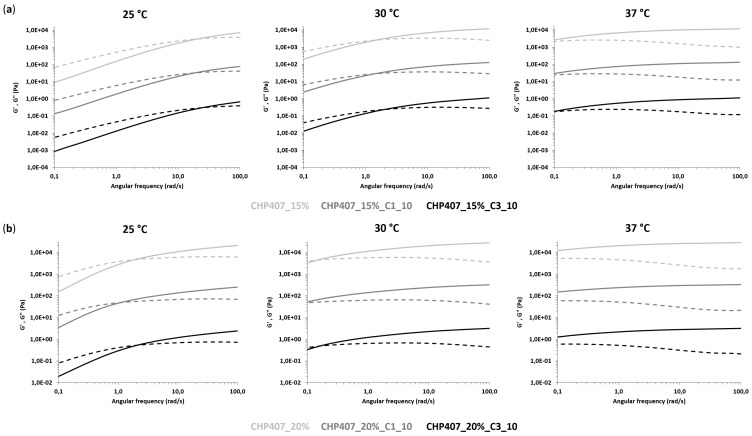
Trends of G’ (continuous line) and G’’ (dashed line) moduli as a function of angular frequency for CHP407 formulations at (**a**) 15% *w/v* and (**b**) 20% *w/v* as such and upon addition of C1 and C3 particles at 10 mg/mL concentration. G′ and G″ values of C1- and C3-loaded hydrogels were divided by a factor of 100 and 10,000, respectively.

Frequency sweep tests performed at 25, 30 and 37 °C provided information on the progressive evolution of hydrogel mechanical properties and transition states from sol to gel during the increase of temperature. Figure 9 reports the trends of G’ and G’’ as a function of angular frequency, as measured at the three tested temperatures. Irrespective of sample composition, the angular frequency range showing G’ values higher than G’’ ones increased with increasing temperature, thus proving the progressive transition of the systems from the sol to the gel phase. As a consequence, the G’/G’’ crossover frequency (ω_G’/G’’_cross_, Table 4) value, which is a reference parameter marking the transition from the typical behavior of solutions to that of gels, decreased with the increasing temperature.

**Figure 10:**
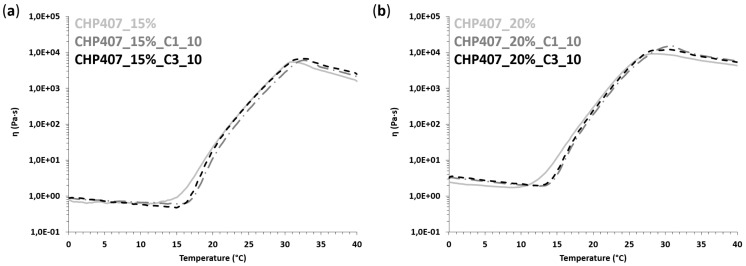
Trends of viscosity (η) as a function of temperature for CHP407 hydrogels at (**a**) 15% *w/v* and (**b**) 20% *w/v* as such and upon addition of C1 and C3 particles at 10 mg/mL concentration.

At 15% *w/v* CHP407 concentration, both purely polymeric and hybrid samples behaved mainly as solutions at 25 °C, becoming clearly biphasic at 30 °C and finally incipient gels at 37 °C. A similar trend was observed at higher polymer concentrations, but the transition from the sol to the gel state began at lower temperatures. Indeed, samples were mostly in the gel phase starting from 30 °C, in accordance with the increased CHP407 content within the systems and our previous findings on similar sol-gel systems [44]. At 37 °C, all formulations were in the gel phase (i.e., ω_G’/G’’_cross_ lower than 0.1 rad/s); however, none of the samples achieved a complete gel development, although those based on CHP407 at 20% *w/v* concentration exhibited a higher degree of network organization with respect to 15% *w/v* concentrated formulations. A further confirmation of hydrogel progressive gelation was provided by the temperature-dependent increase in the delta between G’ and G’’ measured at 100 rad/s (Table 4). In accordance with previous findings from strain sweep tests, the embedding of C1 into CHP407 sol-gel systems turned out to exert more relevant effects on the sol-to-gel transition kinetics compared to C3. Indeed, at each analyzed temperature and CHP407 concentration, the embedding of C1 induced a slight decrease in the measured ω_G’/G’’_cross_ that suggested a quicker gelation of C1-embedding formulations compared to CHP407 sol-gel systems as such. Differently, in the case of C3 particles, the sol-to-gel transition of the hybrid formulations seemed to get slightly slower compared to the control samples (i.e., purely polymeric formulations). The accelerating effect of gelation due to the formed hydrogen bonds was probably down-regulated in C3-containing samples due to their completely different geometrical properties (i.e., shape, size, exposed surface area) compared to C1. Finally, the temperature-driven sol-to-gel transition was investigated through rheological temperature ramp tests, which measured the trend of viscosity (η) as a function of temperature within the range of 0 to 40 °C (Figure 10).

As is typical of fluid systems, sample viscosity initially decreased with the increasing temperature until a minimum value was reached, which marked the beginning of the sol-to-gel transition. Thus, the temperature at the minimum viscosity can be identified as the onset temperature of gelation (T_onset_, Table 5).

Starting from T_onset_, viscosity quickly increased with increasing temperature until the occurrence of gel fracture phenomena as a consequence of their incapability to withstand the continuous strain rate they were subjected to [44]. Irrespective of their composition, all samples, both purely polymeric and hybrid formulations, exhibited a similar trend of viscosity vs. temperature. However, when OMCs were embedded into CHP407 hydrogels, an increase in T_onset_ was observed, which suggested that particle embedding partially slowed down the process of the chain arrangement into micelles and the onset of their organization into a gel network. However, despite this difference in the beginning of gelation, both C1- and C3-embedding systems easily recovered this delay, quickly overcoming their viscosity trends to those of the control samples (CHP407-based sol-gel systems at 15 and 20% *w/v* concentration). As expected, the loading of the particles into the polymeric vehicle phase induced a slight increase in system initial viscosity, defined as the measured value of viscosity at 0 °C (Table 5).

### 3.4. Characterization of Hybrid Sol-Gel Systems Based on Thermosensitive PEU Hydrogels and OMCs Loaded with Ibuprofen

Whereas previous paragraphs were devoted to the investigation of the effects of OMC embedding on the gelation potential of CHP407 sol-gel systems, this section of the work will concentrate on the assessment of potential effects induced by IBU-loading into OMCs before their encapsulation into the polymeric vehicle phase. Finally, IBU release profile from the hybrid formulations will be characterized and compared to the release profile of the drug from CHP407 hydrogels as such.

#### 3.4.1. Rheological Characterization

**Figure 11:**
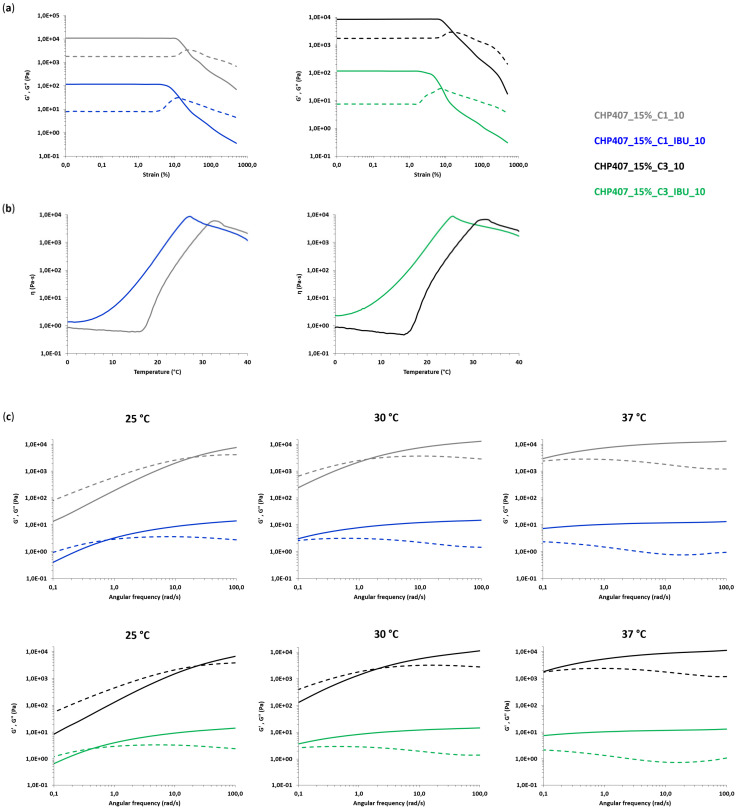
Rheological characterization of CHP407-based hydrogels at 15% *w/v* concentration embedding C1 particles as such and loaded with IBU, or C3 particles as such or loaded with IBU. OMCs were loaded into the hydrogels at 10 mg/mL concentration. (**a**) Trends of G’ (continuous line) and G’’ (dashed line) moduli as a function of applied deformation for CHP407 hydrogels loaded with C1 and C1-IBU particles (left), or C3 and C3-IBU particles (right). G′ and G″ values of C1-IBU- and C3-IBU-loaded hydrogels were divided by a factor of 100. (**b**) Trends of viscosity (η) as a function of temperature for CHP407 hydrogels loaded with C1 and C1-IBU particles (left), or C3 and C3-IBU particles (right). (**c**) Trends of G’ (continuous line) and G’’ (dashed line) moduli as a function of angular frequency for CHP407 hydrogels loaded with C1 and C1-IBU particles (top), or C3 and C3-IBU particles (bottom). G′ and G″ values of C1-IBU and C3-IBU-loaded hydrogels were divided by a factor of 1000.

A complete rheological characterization was conducted on both CHP407_15%_C1_IBU_10 and CHP407_15%_C3_IBU_10 through strain sweep, frequency sweep and temperature ramp tests. The previously commented results on CHP407_15%_C1_10 and CHP407_15%_C3_10 were used as references to evaluate any potential contribution of IBU-loading into OMCs on the overall gelation properties of drug-releasing hybrid formulations based on CHP407 and IBU-OMCs. Figure 11 summarizes the results of the performed characterizations by plotting the measured characteristic curves of IBU-OMC-loaded sol-gel systems and their corresponding control formulations (i.e., CHP407_15%_C1_10 and CHP407_15%_C3_10). Instead, the characteristic parameters extrapolated from rheological curves are collected in Table 6. Plotting of CHP407_15%_C1_IBU_10 and CHP407_15%_C3_IBU_10 rheological curves together with those of their reference samples into the same Cartesian plane immediately highlighted the existence of evident differences between IBU-OMC-loaded sol-gel systems and hydrogels embedding OMCs as such. Strain sweep tests (Figure 11a) evidenced a significant reduction in resistance to applied deformation for both CHP407_15%_C1_IBU_10 and CHP407_15%_C3_IBU_10 with respect to their reference samples (γ_L_ value decreased of 55–60%). As expected, this decrease in the critical deformation corresponding to the onset of fracture phenomena within the gels was accompanied by a decrease in YS of around 45–55%. The decrease in mechanical resistance of IBU-OMC-loaded gels could be explained by the increase in the G’-G’’ gap within the LVE region, which suggests the achievement of a more developed and organized gel network for these formulations with respect to CHP407_15%_C1_10 and CHP407_15%_C3_10 control samples (e.g., Δ_G’-G’’_ increased from 6770 Pa in CHP407_15%_C3_10 to 10,717 Pa in CHP407_15%_C3_IBU_10). The hypothesis of an increased gel network development for IBU-OMC-loaded gel systems, compared to formulations containing OMCs as such, was further corroborated by frequency sweep test results (Figure 11c), which evidenced significantly lower G’/G’’ crossover frequency values (ω_G’/G’’_cross_) for both CHP407_15%_C1_IBU_10 and CHP407_15%_C3_IBU_10 compared to CHP407_15%_C1_10 and CHP407_15%_C3_10, at each analyzed temperature (Table 6). Indeed, at 25 °C, ω_G’/G’’_cross_ decreased of about 95–98% upon embedding of IBU-OMCs instead of plain OMCs into CHP407 hydrogels, becoming lower than 0.1 rad/s starting from 30 °C. Furthermore, temperature ramp test results highlighted that the gelation process of both CHP407_15%_C1_IBU_10 and CHP407_15%_C3_IBU_10 began at very low temperatures (Figure 11b). This did not allow a clear definition of the onset temperature of gelation (T_onset_, Table 6), since the initial typical decreasing trend of viscosity vs. temperature is missing from the measured curves. As a consequence of this early beginning of the sol-to-gel transition, both CHP407_15%_C1_IBU_10 and CHP407_15%_C3_IBU_10 exhibited significantly higher viscosity values compared to CHP407_15%_C1_10 and CHP407_15%_C3_10 within the whole analyzed temperature range, as highlighted in Table 6, which reports viscosity values measured at 0 and 25 °C (η_0 °C_ and η_25 °C_, respectively). Accordingly, due to the increased mechanical properties of the gels and their decreased capability to withstand applied mechanical stress, melt fracture phenomena occurred in CHP407_15%_C1_IBU_10 and CHP407_15%_C3_IBU_10 at lower temperatures compared to CHP407_15%_C1_10 and CHP407_15%_C3_10, thus proving that the mechanical response, which resulted in gel failure, was achieved at lower temperatures.

Altogether, the measured rheological behavior of IBU-OMC-loaded sol-gel systems evidenced a key role exerted by IBU infiltrated into the mesoporous carbons in the gelation properties of drug-releasing hybrid formulations. By combining the behavior observed in the present work with our recently published data on the rheological properties of CHP407 sol-gel systems embedding IBU as a free molecule [52], we can hypothesize the role exerted by the drug in the gelation process. In detail, we have recently demonstrated at both the macro- and micro-scales that ibuprofen molecules tend to be integrated into the hydrophobic core of micelles during their formation process, leading to: (a) the achievement of the critical micellar volume required for the onset of gelation at lower temperatures, and (b) faster gelation kinetics and a more brittle gel network compared to samples not-containing IBU molecules. Thus, based on these previous results, we can suppose that during system preparation, a fraction of encapsulated IBU molecules leaks out of the mesopores and due to the hydrophobic nature of the drug, this fraction is encapsulated in the cores of the adjacent micelles leading, in the end, to the observed acceleration in gelation kinetics and improved mechanical properties.

#### 3.4.2. In Vitro Drug Release Tests

**Figure 12:**
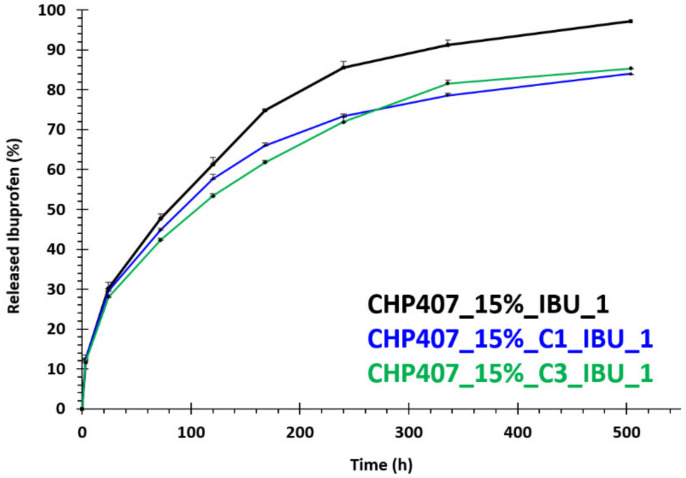
Ibuprofen release profile (%) from CHP407-based hydrogels at 15% *w/v* concentration embedding C1_IBU particles, C3_IBU particles and IBU as such.

Ibuprofen release profile was studied from IBU-OMC-containing CHP407 hydrogels (CHP407_15%_C1_IBU_1 and CHP407_15%_C3_IBU_1) at 15% *w/v* polymer concentration and 1 mg/mL OMC content corresponding to 1 mg/mL IBU concentration (i.e., 2 mg/mL IBU-OMC concentration). Towards this aim and for comparison purposes, IBU release profile was also studied from CHP407 hydrogels (15% *w/v*) embedding IBU as such at 1 mg/mL concentration (CHP407_15%_IBU_1). Figure 12 reports the IBU release profiles measured from the investigated formulations up to 3 weeks incubation in aqueous medium (i.e., PBS) at physiological temperature.

While CHP407_15%_IBU_1 samples completely released their cargo within the three weeks of analysis, formulations containing IBU-OMCs delivered approximately 85% of their payload within the same time frame. These results clearly indicate the capability of mesoporous carbons to effectively work as drug reservoirs enabling a sustained and prolonged release of bioactive agents over time. In contrast with our previous work in which we characterized CHP407 hydrogels (15% *w/v*) carrying IBU-loaded mesoporous bioactive glasses (MBGs) [35], the release profile of ibuprofen from both CHP407_15%_C1_IBU_1 and CHP407_15%_C3_IBU_1 did not achieve a plateau value, but a slowly increasing trend was still evident after 3 weeks of incubation in aqueous medium. Indeed, a distinctive difference from silica-based mesoporous bioactive glasses, is that OMCs did not exhibit pore occlusion phenomena, which were most likely responsible for the incomplete ibuprofen release observed in CHP407 hydrogels embedding IBU-loaded MBGs. No significant differences in IBU release profile were observed between CHP407_15%_C1_IBU_1 and CHP407_15%_C3_IBU_1, suggesting that the different geometrical features of C1 and C3 did not significantly affect their release potential when embedded into CHP407 sol-gel systems. On the other hand, starting from 3 days incubation time, the amount of drug released from CHP407_15%_IBU became significantly higher compared to that measured for IBU-OMC-embedding gels. This is a further proof that in both CHP407_15%_C1_IBU_1 and CHP407_15%_C3_IBU_1 the drug had to pass through a double release barrier, namely the mesoporous carbon framework and the gel network, before reaching the release medium. Conversely, for shorter incubation times, irrespective of their compositions, all the samples behaved in a similar way, releasing approximately 30% of their cargo within the first 24 h incubation in an aqueous medium. This similarity in the initial amount of released drug indirectly proved our previous hypothesis on the rheological behavior of IBU-OMC-embedding CHP407 gels. Indeed, as IBU-OMCs immediately released an aliquot of the loaded drug upon dispersion in the aqueous phase and encapsulation into the hydrogels, gels loaded with them behaved in a similar way as formulations containing ibuprofen as such in the initial time interval of the release tests.

## 4. Conclusions

The design of drug delivery systems that carry a huge amount of active molecules and release them to a target biological compartment according to a controlled and sustained profile represents an overwhelming advancement in the biomedical field. Indeed, such an approach overcomes the main drawbacks of traditional drug administration routes (e.g., oral and systemic administration), limiting the need for multiple administrations and opening the way to the possibility to set up personalized therapies. With this perspective in mind, in this work we reported on the possibility to design hybrid injectable thermosensitive sol-gel formulations. To this end, we combined ordered mesoporous carbons and thermosensitive poly(ether urethane)-based sol-gel systems, relying on the well-known high drug loading capability, biocompatibility and chemical stability of the former [20] and the improved gelation, mechanical properties and residence time of the latter compared to commercial P407-based hydrogels [44]. OMCs with rod-like and spherical shapes were successfully synthesized and completely filled with ibuprofen (100% loading yield, 1:1 carbon/IBU weight ratio). Meanwhile, a high molecular weight poly(ether urethane) ($\bar{M_{n}}$ 72 kDa, D 1.7) was obtained starting from P407, HDI and CDM as building blocks. The infiltration of ibuprofen in the pores of the OMCs was first assessed through DSC analysis, which evidenced the absence of the crystalline drug for both particle types. The process of OMC loading into the hydrogel vehicle phase did not negatively affect the gelation potential of the investigated formulations. Interestingly, the gelation process of the hydrogels turned out to be influenced by the OMC surface decoration with the surfactant SDS. Specifically, its hydrophilic heads probably took an active part in the sol-to-gel transition through the formation of hydrogen bonds with CHP407 chains, thus allowing OMCs to act as nodes within the gel network. In addition, OMC geometrical features also appeared to affect gel properties, with smaller spherical C1 particles better integrating within CHP407 gels. The high yield of IBU encapsulation into OMCs allowed the loading of a high drug content into the hydrogels through the embedding of a small amount of OMCs. Furthermore, OMCs turned out to effectively act as drug reservoirs, enabling a prolonged and sustained release of their cargo over time. Indeed, while hydrogels containing ibuprofen as such completely released it within 3 weeks, OMC-loaded systems delivered ca. the 85% of their payload within the same time interval. Such features make the formulations developed in this work able to perfectly address the previously mentioned demands, thus opening the way for their potential exploitation in the biomedical field to treat a wide range of pathological states (e.g., chronic skin wounds, delayed bone healing, cancer, damaged cartilage) upon a proper selection of the therapeutic agents to load. However, in their current formulation, the here-characterized hybrid sol-gel systems would not be suitable for application in photothermal therapy due to the LCGT behavior of CHP407 poly(ether urethane)-based aqueous solutions. To make our platform suitable for this application, PEU LEGO-like structure could be exploited to include in the polymer backbone proper functional groups (e.g., thiols, amines, aldehyde, carboxyl groups), which will allow a secondary stabilization of the thermo-induced gel network through the formation of chemical crosslinks making the resulting system unresponsive to further temperature changes. Finally, by exploiting both the micellar nature of the hydrogels and the mesoporous framework of OMCs, formulations releasing a cocktail of compounds, each according to a specific time schedule, could be developed for advanced therapeutic applications.

## Figures and Tables

**Table 1:** Compositional information and acronyms of the formulations designed in this work.

CHP407 (% *w/v*)	OMC		Acronym
	Type	Concentration (mg/mL)	
15	-	-	CHP407_15%
15	C1	10	CHP407_15%_C1_10
15	C3		CHP407_15%_C3_10
15	C1_IBU		CHP407_15%_C1_IBU_10
15	C3_IBU		CHP407_15%_C3_IBU_10
20	-	-	CHP407_20%
20	C1	10	CHP407_20%_C1_10
20	C3		CHP407_20%_C3_10

**Table 2:** Pore properties (specific surface area S_BET_, total pore volume TPV, micropore volume V_micro_, mesopore volume V_meso_ and pore width) of pristine and IBU-loaded C1 and C3.

Sample Acronym	S_BET_ (m^2^/g)	TPV (cm^3^/g) ^1^	V_micro_ (cm^3^/g)	V_meso_ (cm^3^/g)	Pore Width (nm)
C3	1244	1.14	0.18	0.96	4.9
C1	1956	1.19	0.24	0.95	3.2
C3_IBU	11	0.02	0.00	0.01	-
C1_IBU	22	0.07	0.00	0.06	-

^1^ at *p*/*p*_0_ = 0.90 (for pores with diameters < ~20 nm).

**Table 3:** Characteristic parameters of strain sweep tests: γ_L_, critical strain value corresponding to the limit of the Linear Viscoelastic (LVE) region; YS, value of shear stress at the maximum of loss modulus; and Δ_G’-G’’_, delta between G’ and G’’ within the LVE region.

SAMPLE	γ_L_ (%)	Δ_G’-G’’_ (Pa)	YS (Pa)
CHP407_15%	4.5	8109	407
CHP407_15%_C1_10	11.6	9008	1110
CHP407_15%_C3_10	6.2	6770	675
CHP407_20%	2.8	22,860	921
CHP407_20%_C1_10	7.3	26,649	1940
CHP407_20%_C3_10	3.0	24,809	920

**Table 4:** Characteristic parameters of frequency sweep tests: ω_G’/G’’_cross_, crossover frequency between G’ and G’’ trends; Δ_G’-G’’_100rad/s_, difference between G’ and G’’ values measured at 100 rad/s.

SAMPLE	25 °C		30 °C		37 °C	
	ω_G’/G’’_cross_ (rad/s)	Δ_G’-G’’_100rad/s_ (Pa)	ω_G’/G’’_cross_ (rad/s)	Δ_G’-G’’_100rad/s_ (Pa)	ω_G’/G’’_cross_ (rad/s)	Δ_G’-G’’_100rad/s_ (Pa)
CHP407_15%	20.8	3140	1.5	9190	- *	10,812
CHP407_15%_C1_10	18.6	3610	1.4	10,130	- *	11,990
CHP407_15%_C3_10	23.4	2870	2.1	8420	- *	10,130
CHP407_20%	1.9	14,860	0.1	24,060	- *	26,710
CHP407_20%_C1_10	1.1	18,190	- *	28,230	- *	31,250
CHP407_20%_C3_10	2.1	16,670	0.2	26,940	- *	29,900

* ω_G’/G’’_cross_ < 0.1 rad/s.

**Table 5:** Characteristic parameters of temperature ramp tests: T_onset_, onset temperature of the gelation process; η_0 °C_, viscosity measured at 0 °C.

SAMPLE	T_onset_ (°C)	η_0 °C_ (Pa∙s)
CHP407_15%	12.4	0.8
CHP407_15%_C1_10	15.7	0.9
CHP407_15%_C3_10	15.4	0.9
CHP407_20%	9.7	2.4
CHP407_20%_C1_10	13.7	3.1
CHP407_20%_C3_10	13.4	3.2

**Table 6:** Characteristic parameters of rheological strain sweep, frequency sweep and temperature ramp tests performed on CHP407_15%_C1_IBU_10 and CHP407_15%_C3_IBU_10.

Rheological Parameter	CHP407_15%_C1_IBU_10	CHP407_15%_C3_IBU_10
Strain sweep test results		
**γ_L_ (%)**	4.5	6.2
**Δ_G’-G’’_ (Pa)**	10,874	10,717
**YS (Pa)**	607	308
Frequency sweep test results		
**ω_G’/G’’_cross_ (rad/s) 25 °C**	0.8	0.4
**ω_G’/G’’_cross_ (rad/s) 30 °C**	- *	- *
**ω_G’/G’’_cross_ (rad/s) 37 °C**	- *	- *
Temperature ramp test results		
**T_onset_ (°C)**	- **	- **
**η_0 °C_ (Pa∙s)**	1.3	2.3
**η_25 °C_ (Pa∙s)**	4394	7914

* ω_G’/G’’_crossover_ < 0.1 rad/s. ** out of the analyzed temperature range.
